# Relationship between inherited genetic variation and survival from colorectal cancer stratified by tumour location

**DOI:** 10.1038/s41598-024-77870-0

**Published:** 2025-01-18

**Authors:** Christopher Wills, Katie Watts, Amy Houseman, Timothy S. Maughan, David Fisher, Nada A. Al-Tassan, Richard S. Houlston, Valentina Escott-Price, Jeremy P. Cheadle

**Affiliations:** 1 Division of Cancer and Genetics, School of Medicine, Cardiff University, Heath Park, Cardiff, CF14 4XN UK; 2 CRUK/MRC Oxford Institute for Radiation Oncology, University of Oxford, Roosevelt Drive, Oxford, OX3 7DQ UK; 3 MRC Clinical Trials Unit, University College of London, 125 Kingsway, London, WC2B 6NH UK; 4 Department of Genetics, King Faisal Specialist Hospital and Research Center, P.O. Box 3354, 11211 Riyadh, Saudi Arabia; 5 Division of Genetics and Epidemiology, The Institute of Cancer Research, London, SW7 3RP UK; 6 Institute of Psychological Medicine and Clinical Neurosciences, School of Medicine, Cardiff University, Hadyn Ellis Building, Maindy Road, Cardiff, CF24 4HQ UK

**Keywords:** Colorectal cancer, Survival, Tumour location, Germline variation, Prognostic markers, Cancer genomics

## Abstract

The location of a patient’s colorectal cancer (CRC) influences their outcome but inherited factors may also be involved. We studied 1899 patients with advanced CRC (514 had proximal colonic, 493 distal colonic and 892 rectal tumours) and carried out genome-wide association studies for survival. Single nucleotide polymorphisms (SNPs) suggestive of association (*P* < 1.0 × 10^–5^) were tested for replication in 5078 CRC patients from the UK Biobank. We investigated the relationship between Phosphatidylinositol 4-Kinase Type 2 Beta (*PI4K2B*) expression in colorectal tumours and survival in 597 patients from The Human Protein Atlas (THPA). We also analysed 3 SNPs previously associated with survival by anatomical site. We found that SNPs at 54 independent loci were suggestive of an association with survival when stratified by tumour location. rs76011559 replicated in patients with proximal tumours (COIN, COIN-B and UK Biobank combined Hazard Ratio [HR] = 1.53, 95% Confidence Intervals [CI] = 1.19–1.86, *P* = 7.5 × 10^–7^) and rs12273047 replicated in patients with rectal tumours (combined HR = 1.27, 95% CI = 1.09–1.46, *P* = 4.1 × 10^–7^). In gene analyses, *PI4K2B* associated with survival in patients with distal cancers (*P* = 2.1 × 10^–6^) and increased *PI4K2B* expression in colorectal tumours was associated with improved survival (*P* = 9.6 × 10^–5^). No previously associated SNPs were replicated. Our data identify novel loci associated with survival when stratified by tumour location.

## Introduction

Proximal and distal colonic cancers have distinct clinicopathological and molecular features, reflective of their embryological origin and biology^1,2^. Proximal colonic cancers are frequently *KRAS*^3,4^ and *BRAF*^1,4^ mutated, have microsatellite instability and a CpG island methylator phenotype^5^. They are more common in women and older patients, and while having a poorer prognosis, tend have a better response to 5-fluorouracil chemotherapy^2^. Distal cancers are typified by chromosomal abnormalities and aneuploidy^6^. Rectal cancers have higher rates of locoregional relapse, a preference for lung metastases and a lower frequency of *KRAS* and *BRAF* mutations^7–9^.

The prognosis for patients with the same stage of colorectal cancer (CRC) can vary and in addition to clinicopathological features and somatic mutations it is being recognised that germline variation also influences outcome. Our recent work identified germline variants associated with survival in patients with advanced CRC from the clinical trials COIN and COIN-B^10^. Given the inherent differences in the pathobiology of proximal and distal cancers, here we report on the impact of germline variation on CRC prognosis by tumour anatomical site.

## Results

### Our study cohort

**Fig. 1:**
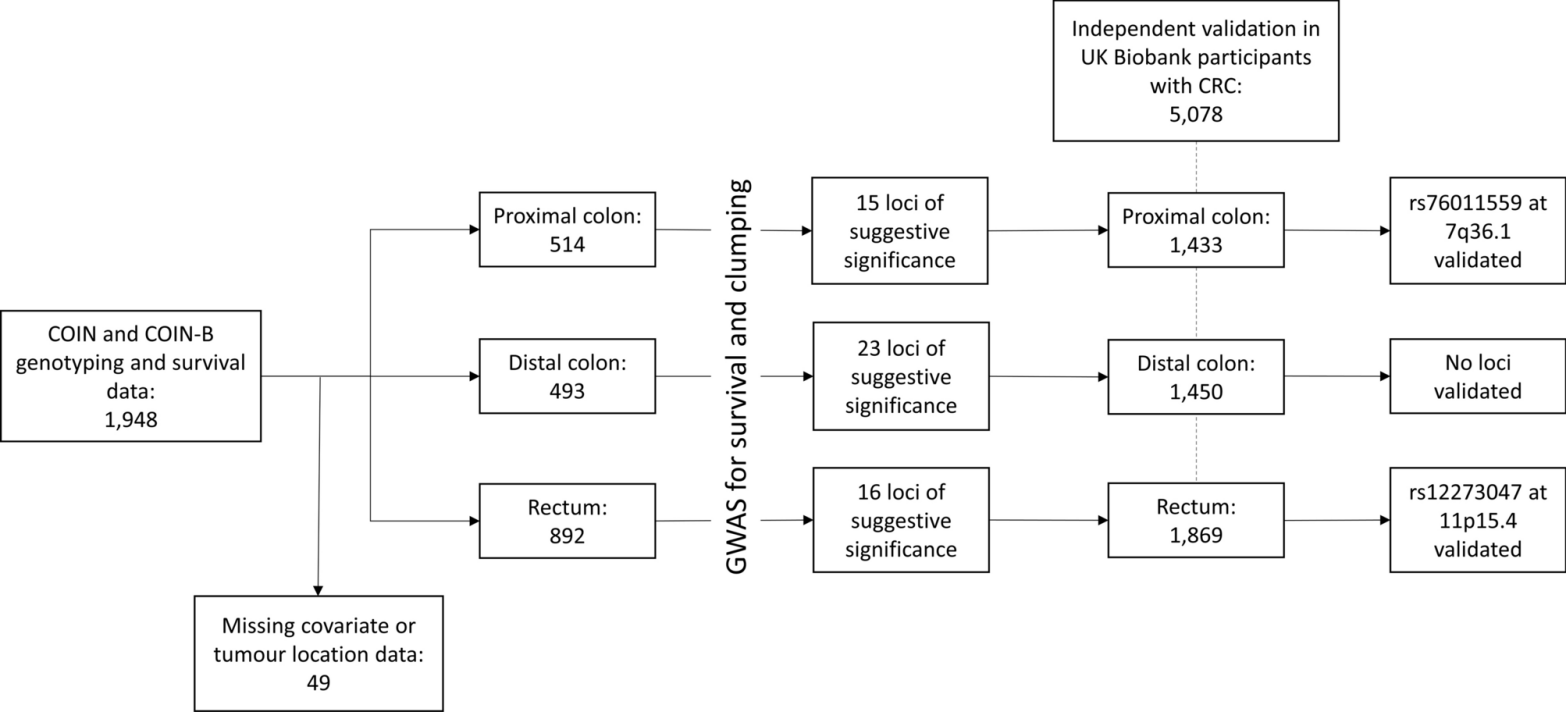
Flow diagram depicting the genetic and survival analyses of patients from COIN and COIN-B by primary tumour location. 514 patients had primary tumours in the proximal colon, 493 in the distal colon and 892 in the rectum. Lead single nucleotide polymorphisms from independent loci suggestive of association with survival were tested for replication in participants from the UK Biobank with proximal colon (n = 1433), distal colon (n = 1450) and rectal cancers (n = 1869), respectively.

We studied 2244 unrelated patients with metastatic or locally advanced CRC recruited into the MRC clinical trials COIN^11^ and COIN-B^12^. After quality control (QC), whole genome genotyping and survival data were available on 1948 patients. We assigned patients to groups by location of their primary tumour (for 49 patients this data was missing, leaving n = 1899)^13^. Proximal tumours—those within the hepatic flexure, transverse colon, cecum and ascending colon (514 patients, 413 with events); Distal tumours—those within the descending colon, sigmoid colon and splenic flexure (n = 493 patients, 358 with events); Rectal tumours—those within the rectosigmoid junction and rectum (892 patients with 645 events) (Fig. 1).

Patients with proximal CRCs had a higher frequency of *KRAS* (39.1%) and *BRAF* (16.0%) mutations and worse prognosis (median survival 397 days) compared to patients with distal (25.6%, 4.3% and 514 days, all* P* < 1.0 × 10^–4^, respectively) and rectal cancers (33.3%, *P* = 1.2 × 10^–2^; 4.1%, *P* < 1.0 × 10^–4^ and 520 days, *P* < 1.0 × 10^–4^, respectively) (Table 1).

**Table 1:** Clinicopathological features of COIN and COIN-B patients by tumour site.

Clinicopathological factor		Proximal tumour		Distal tumour		Rectum		*P*
		(n = 514)		(n = 493)		(n = 892)		
		n	%	n	%	n	%	
Sex	Male	307	59.7	312	63.3	625	70.1	2.2 × 10^–4^
	Female	207	40.3	181	36.7	267	29.9	
Age	Median (years)	65	–	64	–	63	–	–
Overall survival	Median days (95% CI)	397 (359–444)	–	514 (471–556)	–	520 (496–581)	–	< 1.0 × 10^–4^
WHO performance status	0	216	42.0	209	42.4	459	51.5	1.3 × 10^–3^
	1	251	48.8	249	50.5	375	42.0	
	2	47	9.1	35	7.1	58	6.5	
Status of primary tumour	Resected	316	61.5	270	54.8	421	47.2	< 1.0 × 10^–4^
	Unresected	198	38.5	223	45.2	471	52.8	
Timing of metastases	Metachronous	136	26.5	119	24.1	311	34.9	< 1.0 × 10^–4^
	Synchronous	378	73.5	374	75.9	581	65.1	
Type of metastases	Liver only	86	16.7	151	30.6	185	20.8	< 1.0 × 10^–4^
	Liver plus others	272	52.9	255	51.7	474	53.3	
	Non-liver	156	30.4	87	17.6	231	26.0	
Number of metastatic sites	0	0	0.0	0	0.0	2	0.2	0.23
	1	175	34.0	196	39.8	310	34.8	
	2	200	38.9	181	36.7	367	41.1	
	≥ 3	139	27.0	116	23.5	213	23.9	
*KRAS* status	Mutated	201	39.1	126	25.6	297	33.3	< 1.0 × 10^–4^
	Wild-type	224	43.6	283	57.4	453	50.8	
	n/k	89	17.3	84	17.0	142	15.9	
*NRAS* status	Mutated	16	3.1	20	4.1	30	3.4	0.66
	Wild-type	397	77.2	373	75.7	699	78.4	
	n/k	101	19.6	100	20.3	163	18.3	
*BRAF* status	Mutated	82	16.0	21	4.3	37	4.1	< 1.0 × 10^–4^
	Wild-type	332	64.6	373	75.7	695	77.9	
	n/k	100	19.5	99	20.1	160	17.9	
*PIK3CA* status	Mutated	62	12.1	45	9.1	79	8.9	0.065
	Wild-type	308	59.9	315	63.9	594	66.6	
	n/k	144	28.0	133	27.0	219	24.6	

Data are n (%) or median. Differences between patients were analysed using a Chi-squared test, Fisher’s exact test (for number of metastatic sites) or log rank test (for overall survival). *Non-liver metastases included those in the lungs, peritoneum and lymph nodes. n/k—not known—some data for somatic mutation status was not known due to the lack of availability of tumour tissue or failed amplification.

### Relationship between germline variation and survival by tumour location

**Fig. 2:**
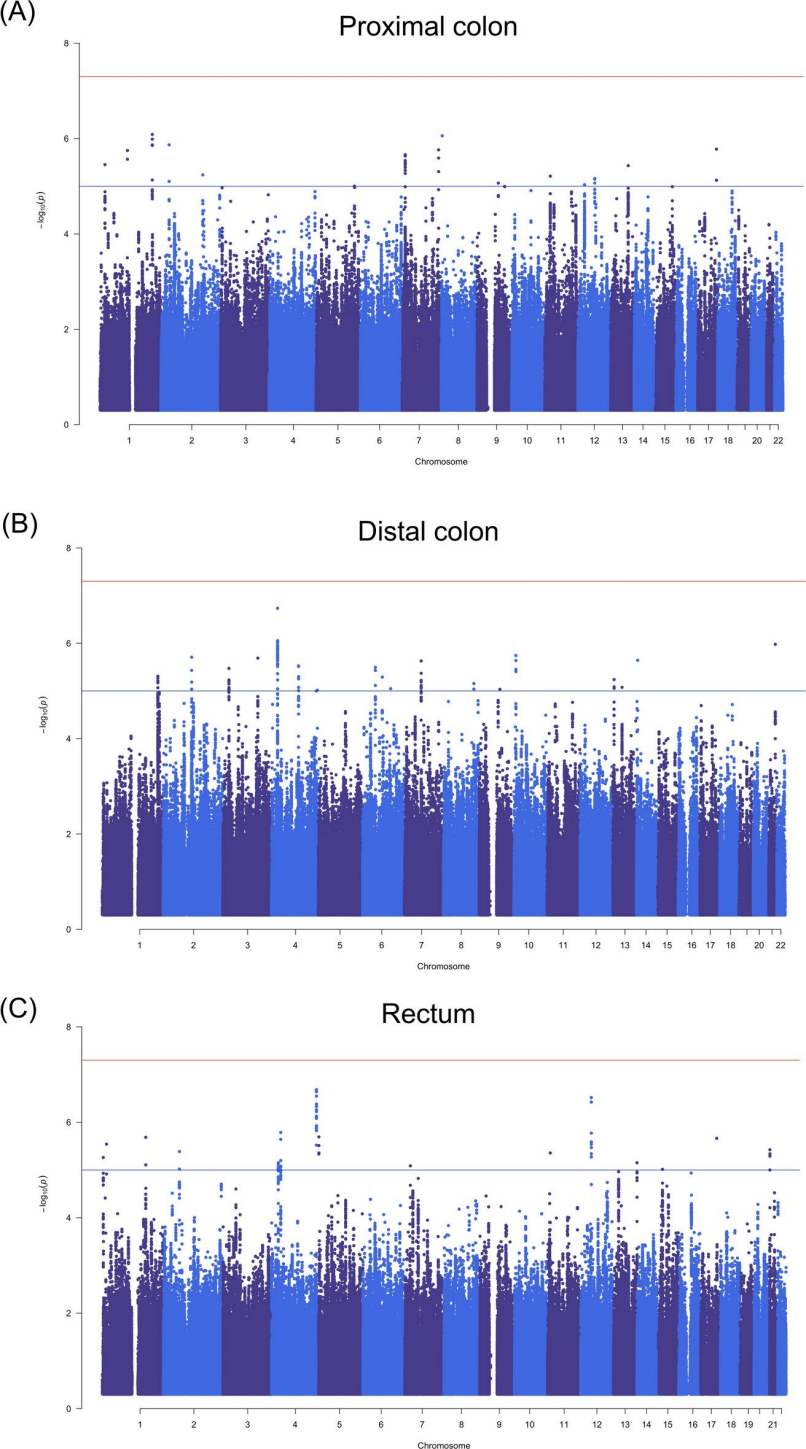
Manhattan plots of single nucleotide polymorphism (SNP) associations with survival in patients from COIN and COIN-B with primary tumours in (**A**) the proximal colon (n = 514), (**B**) the distal colon (n = 493) and (**C**) the rectum (n = 892). SNPs are ordered by chromosome position and plotted against the −log_10_(*P*) for their association with survival. The red line represents the threshold for genome-wide significance (*P* < 5.0 × 10^–8^) and the blue line is the threshold for suggestive significance (*P* < 1.0 × 10^–5^).

We investigated whether germline single nucleotide polymorphisms (SNPs) were associated with survival when considering the anatomical site of a patient’s primary tumour. Genome-wide survival analyses of patients from COIN and COIN-B were stratified by primary tumour location using the first five principal components as covariates, which explained 78–80% of the total variance for previously established prognostic factors^10^. There was no detectable genomic inflation (lambda = 1.03–1.12). We found that no SNPs passed genome-wide significance (*P* < 5.0 × 10^–8^) for association with survival, regardless of tumour location (Fig. 2).

We did however find that SNPs at 15 independent loci were suggestive of an association with survival in patients with tumours in the proximal colon, 23 loci in those with tumours in the distal colon and 16 loci in those with tumours in the rectum (Fig. 2, Table 2).

**Table 2:** Replication of loci suggestive of association with survival in COIN and COIN-B.

Primary tumour location	SNP	Locus	Minor allele	Genes	COIN and COIN-B			UK Biobank		
					HR	95% CI	*P*	HR	95% CI	*P*
Proximal	rs12062055	1q32.3	G		2.02	1.53–2.67	8.2 × 10^–7^	0.90	0.68–1.19	0.46
	rs4304342	8p23.2	C	*CSMD1*	0.67	0.57–0.79	8.8 × 10^–7^	0.98	0.84–1.13	0.77
	rs62135742	2p22.3	C	*LTBP1*	1.80	1.42–2.29	1.4 × 10^–6^	0.97	0.78–1.20	0.75
	rs147899046*	17q25.3	A	*DNAH17*	1.43	1.23–1.65	1.7 × 10^–6^	1.11	0.97–1.27	0.14
	rs76011559	7q36.1	C		1.78	1.40–2.25	1.7 × 10^–6^	1.31	1.03–1.66	**2.8 × 10**^**–2**^
	rs10857917	1p13.2	G	*LOC643355*	1.44	1.24–1.67	1.8 × 10^–6^	0.97	0.84–1.12	0.67
	rs6460936	7p21.3	C	*TMEM106B, VWDE*	1.57	1.30–1.90	2.2 × 10^–6^	1.00	0.83–1.21	0.99
	rs35955655*	1p36.12	CTA	*CDA, DDOST, MIR6084, PINK1, PINK1-AS*	0.71	0.62–0.82	3.5 × 10^–6^	1.05	0.92–1.19	0.47
	rs1388194	13q31.3	T		0.71	0.62–0.82	3.7 × 10^–6^	0.93	0.81–1.06	0.29
	rs112651521	2q31.1	T	*BBS5, FASTKD1, KLHL41, PPIG*	1.71	1.36–2.16	5.8 × 10^–6^	0.99	0.80–1.24	0.96
	rs1514081	11p14.3	C		0.73	0.63–0.83	6.1 × 10^–6^	0.97	0.85–1.10	0.64
	rs10878838	12q15	T	*LOC100507195*	1.64	1.32–2.03	6.9 × 10^–6^	1.09	0.88–1.35	0.44
	rs148684057	9q21.32	GT	*LOC101927575*	1.72	1.35–2.19	8.6 × 10^–6^	0.98	0.80–1.30	0.89
	rs11048907	12p11.23	T	*ARNTL2, C12orf71, MED21, STK38L, TM7SF3*	1.71	1.35–2.16	9.3 × 10^–6^	1.06	0.86–1.32	0.57
	rs78738433	5q33.3	C	*ADAM19, CYFIP2, NIPAL4*	1.90	1.43–2.52	1.0 × 10^–5^	1.04	0.81–1.33	0.77
Distal	rs313566	4p15.2	A	*ANAPC4, PI4K2B, SEPSECS, SEPSECS-AS1, ZCCHC4*	0.52	0.41–0.67	1.8 × 10^–7^	1.15	0.93–1.42	0.19
	rs2837637*	21q22.2	A	*DSCAM*	1.47	1.26–1.72	1.0 × 10^–6^	1.10	0.96–1.26	0.17
	rs7907707	10p14	C		1.63	1.33–1.99	1.8 × 10^–6^	1.04	0.86–1.26	0.70
	rs10182527	2q14.1	T	*DPP10, DPP10-AS1*	1.44	1.24–1.67	2.0 × 10^–6^	1.08	0.95–1.24	0.24
	rs76041099	3q23	C	*LOC100507389*	2.14	1.57–2.94	2.0 × 10^–6^	0.83	0.61–1.14	0.26
	rs11159167	14q12	G		1.43	1.23–1.67	2.3 × 10^–6^	0.97	0.84–1.12	0.69
	rs117589090	10p14	G		2.08	1.53–2.81	2.3 × 10^–6^	0.89	0.64–0.24	0.50
	rs4718825	7q11.22	G		1.55	1.29–1.87	2.3 × 10^–6^	0.98	0.82–1.17	0.83
	rs7656285	4q25	C	*LRIT3, RRH*	1.42	1.22–1.64	3.0 × 10^–6^	0.93	0.81–1.07	0.34
	rs6921841	6p12.2	A		1.62	1.32–1.98	3.2 × 10^–6^	1.05	0.88–1.26	0.56
	rs10510552	3p24.2	T		1.45	1.24–1.69	3.4 × 10^–6^	0.88	0.76–1.00	0.06
	rs34507557	1q42.13	CT	*CDC42BPA*	1.66	1.34–2.07	4.9 × 10^–6^	1.10	0.91–1.34	0.33
	rs28583014	4q25	A	*EGF, ELOVL6*	1.73	1.37–2.20	5.0 × 10^–6^	0.93	0.74–1.17	0.53
	rs2057331	6q14.1	G	*C6orf7*	1.80	1.40–2.33	5.1 × 10^–6^	0.96	0.75–1.23	0.75
	rs41268739	1q42.13	T	*CDC42BPA*	2.04	1.50–2.78	5.4 × 10^–6^	0.90	0.65–1.25	0.54
	rs9995789	4q25	T	*ELOVL6*	1.52	1.27–1.83	5.6 × 10^–6^	0.98	0.82–1.17	0.84
	rs7319699	13q12.12	G	*TNFRSF19*	1.45	1.24–1.71	5.8 × 10^–6^	1.10	0.95–1.27	0.21
	rs7826050	8q24.13	G	*DERL1*	1.45	1.23–1.70	7.0 × 10^–6^	0.99	0.85–1.15	0.87
	rs11842682	13q21.1	T		1.51	1.26–1.81	8.4 × 10^–6^	0.94	0.79–1.12	0.50
	rs1033393	6q22.1	T		1.57	1.29–1.92	8.9 × 10^–6^	1.02	0.85–1.23	0.80
	rs2796466	9q21.32	T	*TLE1*	1.41	1.21–1.64	9.2 × 10^–6^	0.87	0.76–1.01	0.06
	rs7660386	4q35.2	G		0.66	0.55–0.79	9.6 × 10^–6^	0.95	0.81–1.11	0.51
	rs72702433	4q34.3	G		1.86	1.41–2.44	1.0 × 10^–5^	0.97	0.74–1.30	0.87
Rectal	rs73011737	4q34.3	T		1.68	1.38–2.04	2.1 × 10^–7^	0.97	0.78–1.22	0.82
	rs77984832	12q12	T		1.82	1.45–2.29	3.0 × 10^–7^	0.87	0.67–1.12	0.28
	rs1562098	4p14	T		1.32	1.18–1.48	1.6 × 10^–6^	0.99	0.86–1.13	0.85
	rs10067149	5p15.33	G		1.31	1.17–1.47	2.0 × 10^–6^	1.04	0.92–1.19	0.50
	rs74602176	1q25.2	A	*BRINP2*	1.72	1.38–2.15	2.1 × 10^–6^	0.91	0.69–1.21	0.53
	rs2949938	17q24.2	A	*PITPNC1*	1.69	1.36–2.10	2.2 × 10^–6^	0.98	0.71–1.34	0.90
	rs60453441	1p36.13	G		0.69	0.59–0.81	2.9 × 10^–6^	1.02	0.87–1.20	0.81
	rs2822995	21q11.2	T	*NRIP1*	1.37	1.20–1.56	3.8 × 10^–6^	1.13	0.97–1.33	0.12
	rs268872	2p14	T	*ACTR2*	1.39	1.21–1.60	4.1 × 10^–6^	0.98	0.84–1.15	0.81
	rs12273047	11p15.4	C		1.33	1.18–1.50	4.4 × 10^–6^	1.19	1.03–1.38	**1.6 × 10**^**–2**^
	rs35066664	1p36.32	G		1.69	1.35–2.11	5.5 × 10^–6^	0.98	0.77–1.27	0.90
	rs34529111	4p14	G		1.45	1.24–1.71	6.3 × 10^–6^	1.09	0.90–1.31	0.37
	rs112063020	13q34	AGTTT	*CDC16, UPF3A*	1.31	1.17–1.48	7.0 × 10^–6^	1.07	0.93–1.23	0.36
	rs16878917	4p15.2	A		0.74	0.64–0.84	7.1 × 10^–6^	0.98	0.84–1.14	0.78
	rs113230287	7p15.3	C	*STEAP1B*	1.45	1.23–1.72	8.2 × 10^–6^	0.86	0.71–1.05	0.14
	rs78745358	15q14	A	*C15orf41*	1.63	1.31–2.02	9.7 × 10^–6^	1.01	0.72–1.34	0.93

Independent replication of lead SNPs was carried out using participants from the UK Biobank (UKB) with proximal colon, distal colon and rectal tumours. Tumour location, minor allele, Hazard Ratio, 95% confidence intervals and *P* value are listed for survival (time from trial recruitment to death or end of study for COIN and COIN-B, and time from diagnosis to death or data distribution date for the UKB). rs76011559 replicated in patients with proximal tumours and rs12273047 replicated in patients with rectal tumours (in bold). *rs35955655, rs147899046 and rs2837637 were not available in the UKB and so were replaced with the proxies rs12021613 (1000 genomes project R^2^ = 1 and D’ = 1), rs4969218 (R^2^ = 0.99 and D’ = 1) and rs1012846 (R^2^ = 0.6 and D’ = 1), respectively.

### Independent replication

We sought to independently replicate the lead SNPs at each of these loci in 5078 patients with CRC from the United Kingdom (UK) Biobank (UKB). Patients were stratified according to the location of their tumour; however, for 326 patients there was insufficient information to assign the anatomical site. In total, 1433 (473 with events) had proximal disease, 1450 (420 events) had distal disease and 1869 (495 events) had rectal disease (Fig. 1).

We found that rs76011559 mapping to 7q36.1 (123 kb upstream of *CUL1*) replicated in patients with proximal tumours (Hazard Ratio [HR] = 1.31, 95% Confidence Intervals [CI] = 1.03–1.66, *P* = 2.8 × 10^–2^, Supplementary Fig. 1, Table 2). In the advanced disease setting, patients carrying at least one copy of the minor (C) allele had a median reduction in survival of 121 days compared to patients homozygous for the major (A) allele (Supplementary Fig. 1). The prevalence of *KRAS* and *BRAF* mutations in proximal tumours from carriers of the rs76011559 minor allele (35% and 19%, respectively) was similar to non-carriers (Table 1).

rs12273047 at 11p15.4 replicated in patients with rectal tumours (HR = 1.19, 95% CI = 1.03–1.38, *P* = 1.6 × 10^–2^; Supplementary Fig. 2, Table 2). Patients carrying at least one copy of the minor (C) allele had a median reduction in survival of 132 days compared to patients homozygous for the major (T) allele (Supplementary Fig. 2). No other lead SNPs were replicated (Table 2).

### Gene analyses

**Fig. 3:**
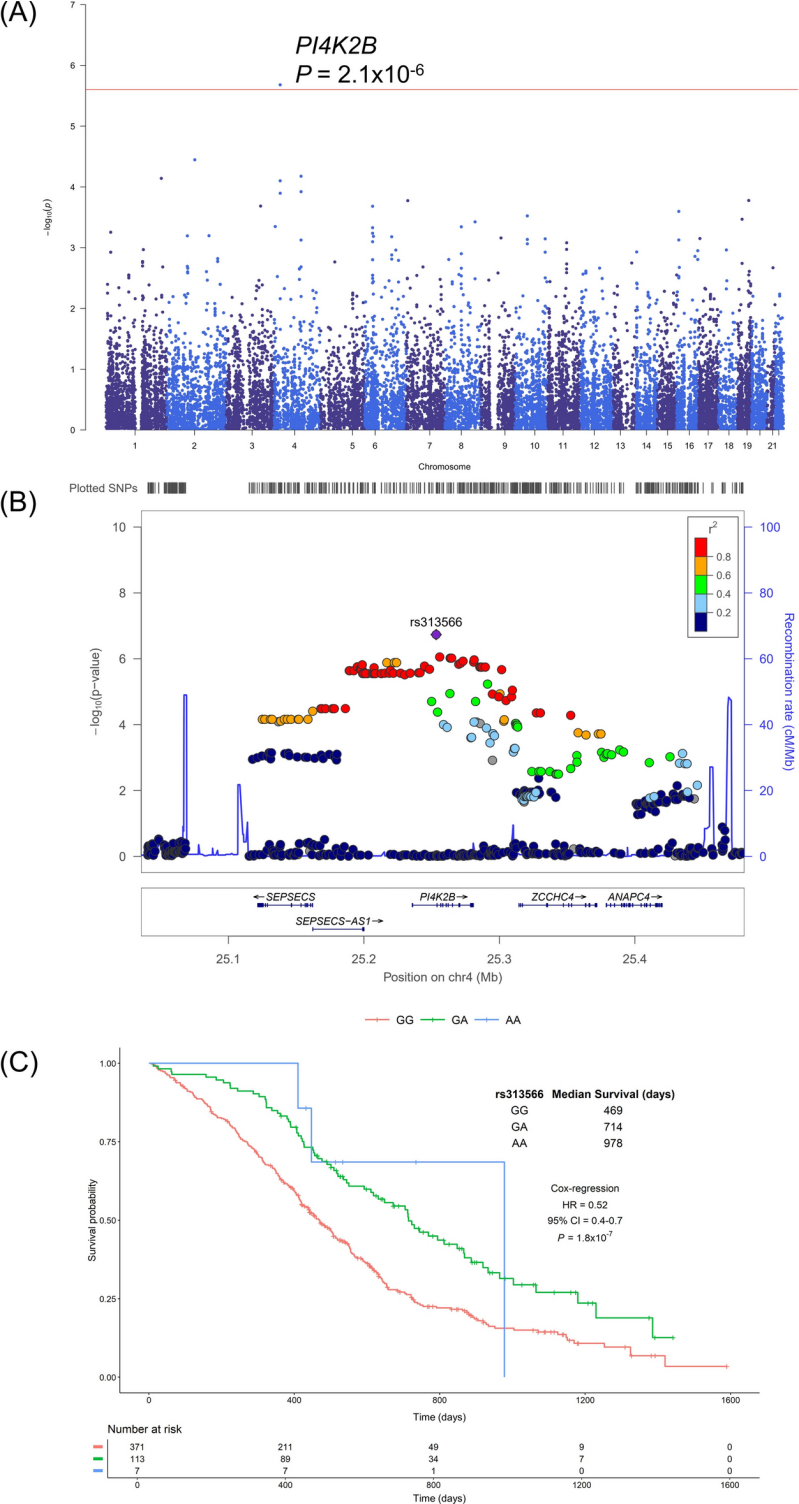
Relationship between gene, genotype and survival in patients from COIN and COIN-B with primary tumours in the distal colon. (**A**) Manhattan plot of gene associations with survival. Genes are ordered by chromosome position and plotted against the −log_10_(*P*) for their association with survival. The red line represents the threshold for genome-wide significance (*P* = 2.5 × 10^–6^). (**B**) Regional locus zoom plot shows results of the analysis for single nucleotide polymorphisms (SNPs) and recombination rates. − log_10_(*P*) (y axis) of the SNPs are shown according to their chromosomal positions (x axis) for an area 200 Kb upstream and downstream of *PI4K2B*. The sentinel SNP (purple) is labelled by its rsID (rs313566). The colour intensity of each symbol reflects the extent of linkage disequilibrium with the sentinel SNP, deep blue (r^2^ = 0) through to dark red (r^2^ = 1.0). Genetic recombination rates, estimated using 1000 Genomes Project samples, are shown with a blue line. Physical positions are based on NCBI build 37 of the human genome. Also shown are the relative positions of genes and transcripts mapping to the region of association. Genes have been redrawn to show their relative positions; therefore, maps are not to physical scale. (**C**) Kaplan–Meier plot of the relationship between rs313566 genotype and survival. Time in days plotted against survival probability for patients homozygous for the major allele (GG) and heterozygous (GA) or homozygous for the minor allele (AA). The number of patients still at risk at each time point is shown beneath.

Gene analyses were performed on the summary statistics from the association analyses to identify genes containing significant numbers of highly associated SNPs. Only Phosphatidylinositol 4-Kinase Type 2 Beta (*PI4K2B*) was significantly associated with survival in COIN and COIN-B patients with distal cancers, beyond the threshold for multiple testing (*P* = 2.1 × 10^–6^; Fig. 3). Patients carrying one copy of the minor (A) allele in the lead SNP, rs313566 in intron 1 of *PI4K2B*, had a median increase in survival of 245 days compared to patients homozygous for the major (G) allele (HR = 0.52, 95% CI = 0.4–0.7, *P* = 1.8 × 10^–7^, Fig. 3). In contrast, rs313566 genotype was not associated with survival in patients with proximal cancers (HR = 1.10, 95% CI = 0.89–1.36, *P* = 0.37, *P*_*Z-test*_ compared to distal cancers = 6.5 × 10^–6^) or those with rectal cancers (HR = 1.16, 95% CI = 0.97–1.39, *P* = 0.09, *P*_*Z-test*_ compared to distal cancers = 1.9 × 10^–7^) (Supplementary Fig. 3).

### Expression analyses

We sought mechanistic understanding of the effect of rs313566 on survival. rs313566 was an expression quantitative trait loci (eQTL) for *PI4K2B* in several cell types (*P* < 3.8 × 10^–5^) with the A-allele associated with increased *PI4K2B* expression (Supplementary Fig. 4).

We sought an association between *PI4K2B* expression levels in colorectal tumours and survival in 597 unrelated patients with CRC from The Human Protein Atlas (THPA). We found that higher *PI4K2B* expression was associated with improved survival (log rank *P* = 9.6 × 10^–5^, Supplementary Fig. 5). This finding was replicated under a linear Cox-proportional hazards model (HR = 0.94, 95% CI = 0.9–1.0, *P* = 7.0 × 10^–3^).

Despite these supportive expression data, we failed to replicate the genetic association between rs313566 and survival in UKB patients with distal (HR = 1.15, 95% CI = 0.93–1.42, *P* = 0.19), proximal (HR = 1.03, 95% CI = 0.84–1.29, *P* = 0.74) or rectal (HR = 1.11, 95% CI = 0.91–1.34, *P* = 0.29) cancers, despite having over 99% power (Supplementary Fig. 3).

### Gene-set analyses

Gene-set analyses were also performed on the summary statistics from the association analyses to identify enriched gene-sets. Four gene-sets (negative regulation of phospholipid biosynthetic process, phosphatidic acid biosynthetic process, 1-acylglycerophosphocholine O-acyltransferase activity and long-term memory) reached significance beyond multiple testing thresholds in patients from COIN and COIN-B with rectal cancers (Supplementary Table 1).

### Meta-analysis of COIN, COIN-B and UKB by tumour location

To increase our power to detect associations, we carried out GWAS for survival in UKB patients by tumour location and meta-analysed the data with COIN and COIN-B. No SNPs reached genome-wide significance although three SNPs were close to this threshold in patients with rectal tumours (rs3980660 at 2q14.3, HR = 0.79, 95% CI = 0.61–0.97, *P* = 2.2 × 10^–7^; rs17237514 at 15q22.2, HR = 0.73, 95% CI = 0.50–0.97, *P* = 2.9 × 10^–7^ and rs12273047 at 11p15.4, HR = 1.27, 95% CI = 1.09–1.46, *P* = 4.1 × 10^–7^; Supplementary Fig. 6). No genes reached genome-wide significance. Three gene-sets (negative regulation of phospholipid biosynthetic process, phosphatidic acid biosynthetic process and positive regulation of response to endoplasmic reticulum stress) reached significance in patients with rectal cancers (Supplementary Table 2).

### Relationship between previously reported prognostic SNPs and tumour location

Three SNPs have previously been associated with CRC-specific survival for cases with tumours in the distal colon (rs698022, HR = 1.48, 95% CI = 1.30–1.69, *P* = 8.47 × 10^–9^) and the proximal colon (rs189655236, HR = 2.14, 95% CI = 1.65–2.77, *P* = 9.19 × 10^–9^ and rs144717887, HR = 2.01, 95% CI = 1.57–2.58, *P* = 3.14 × 10^–8^)^13^. We sought to replicate these observations and analysed these SNPs in patients from COIN and COIN-B.

rs698022 was not replicated despite having 84% power. rs189655236 also failed replication but with more limited power (54%). Although rs144717887 (INFO score = 0.92) was associated with survival in patients with proximal tumours under multivariate analyses (HR = 0.56, 95% CI = 0.32–0.97, *P* = 3.7 × 10^–2^), the direction of effect was opposite to that in the original study and therefore not replicated (Table 3).

**Table 3:** Replication of previously reported SNP associations with survival.

SNP	Allele	Tumour location	N	Events	MAF	INFO	HR	95% CI	*P*
rs189655236	T	Proximal	514	413	0.0078	0.73	0.71	0.31–1.58	0.4
rs144717887	A	Proximal	514	413	0.016	0.92	0.56	0.32–0.97	3.7 × 10^–2^*
rs698022	T	Distal	493	358	0.089	0.83	0.96	0.73–1.26	0.78

Independent replication was carried out using patients from COIN and COIN-B. We had 54, 71 and 84% power to replicate the associations for rs189655236, rs144717887 and rs698022, respectively. Minor allele, tumour location, sample size, number of events, minor allele frequency (MAF) and imputation score (INFO) are shown for each SNP as well as the Hazard ratio (HR), 95% confidence intervals (CI) and *P* value for multivariate analyses. *Although rs144717887 reached statistical significance, the effect was in the opposite direction to the original report and therefore not validated.

## Discussion

We considered the relationship between inherited genetic variation and survival by location of the patient’s CRC. rs76011559 lies upstream of *CUL1* and replicated as a prognostic biomarker in patients with proximal tumours. Proximal tumours from carriers of the rs76011559 minor allele had similar frequencies of *KRAS* and *BRAF* mutations as compared to non-carriers, suggesting that the prognostic effect was independent of somatic mutation status. *CUL1* encodes Cullin1 a member of the Cullin protein family which provides a scaffold for the ubiquitin ligase E3, mediating the degradation of proteins involved in signal transduction, transcription and cell cycle progression. As a consequence, Cullin1 regulates the cell cycle, cell proliferation, invasion, migration and metastasis^14^ and upregulation of Cullin1 in CRC tissue is a negative prognostic biomarker^14–16^. However, rs76011559 was not an eQTL for *CUL1* so further studies are necessary to determine the regulatory mechanism for this SNP.

rs12273047 at 11p15.4 was also replicated in our study in patients with rectal tumours; however, this SNP is intergenic with no clear mechanism of action. It is important to note that the effect sizes associated with both rs12273047 and rs76011559 are modest and therefore unlikely to have direct applications to patient management.

*PI4K2B* was associated with survival in patients with distal cancers, beyond the threshold for multiple testing and the lead SNP rs313566 was not associated with survival in patients with proximal or rectal tumours—suggesting anatomical specificity. We sought further mechanistic understanding of this SNP. rs313566 was an eQTL for *PI4K2B* in several cell types with the A-allele associated with increased expression. Interestingly, we found that higher *PI4K2B* expression in tumour tissue was associated with improved survival in patients with colorectal tumours from THPA. *PI4K2B* encodes a member of the type II PI4 kinase protein family, responsible for overall PI4-kinase activity of the cell and PI4KII beta depletion has been associated with a more invasive phenotype in minimally invasive cell lines^17^. It remains unclear how this function relates to the apparent anatomical specificity that we observed. It should be noted that we failed to replicate the association between rs313566 and survival in UKB patients with distal tumours which may indicate that this was a false positive association, although it may also reflect the heterogeneous stages of cancers in patients from the UKB (whereas in COIN and COIN-B they were all from the advanced disease setting). Further studies are therefore necessary to substantiate our observations.

The gene-sets ‘negative regulation of phospholipid biosynthetic process’ and ‘phosphatidic acid biosynthetic process’ remained significant in our meta-analyses in patients with rectal cancers. Phospholipids have a wide range of physiological functions, including forming the cell membrane, regulating apoptosis and mitochondrial physiology, and phospholipid-derived messenger molecules are involved in intra and extra-cellular signalling. Interestingly, total amount of phospholipids in the cell membrane has been associated with cancer transformation of the cell, with differences in phospholipid composition being predictive of CRC metastases^18^. Phosphatidic acid is an important molecule for the stability and activity of the mTOR complex, a protein kinase that suppresses apoptotic signals in cancer cells^19^. These associations are intriguing given their probable biology and are candidates that warrant further investigation.

## Methods

### Patients and genotyping

Our analyses were based on 2244 unrelated patients with metastatic or locally advanced CRC recruited into the MRC clinical trials COIN (NCT00182715)^11^ and COIN-B (NCT00640081)^12^ who received oxaliplatin and fluoropyrimidine chemotherapy, with or without cetuximab. All patients gave informed consent for bowel cancer research (approved by NHS Research Ethics Committee [04/MRE06/60]) and all methods were performed in accordance with the relevant guidelines and regulations. Since there was no evidence of heterogeneity in overall survival (OS; time from trial randomisation to death or end of trial) between patients when analysed by trial, trial arm, type of chemotherapy received, or cetuximab use, we considered the group as a whole^10^.

DNA was extracted from EDTA-blood samples by conventional methods and genotyped using Affymetrix Axiom Arrays^20^. Prediction of untyped SNPs was carried out with IMPUTE2 v2.3.0^21^ using data from the 1000 Genomes Project as reference^22,23^. Discordant sex, individual and SNP missingness, heterozygosity, relatedness, principal component analysis (PCA), minor allele frequency (MAF) and Hardy Weinberg Equilibrium (HWE) QC steps were performed as previously described^10^. In brief, we excluded SNPs with MAFs < 5%, poor imputation scores (INFO score < 0.8), missingness > 0.02 or HWE exact test *P* < 1.0 × 10^–6^. After QC, genotype data was available on 1950 patients for whom 2 were missing data on survival, leaving 1948 with germline genotyping and survival data.

DNA was extracted from formalin fixed paraffin embedded CRC for 1647 patients (301 tissue samples were not available or were of insufficient quality) and screened for *KRAS* (codons 12, 13 and 61), *NRAS* (codons 12 and 61), *BRAF* (codons 594 and 600) and *PIK3CA* (codons 542, 545, 546 and 1047) mutations using Pyrosequencing and Sequenom technologies^24^. Overall, *KRAS* mutations were identified in 637/1589 (40.1%), *NRAS* mutations in 54/1546 (3.5%), *BRAF* mutations in 143/1554 (9.2%) and *PIK3CA* mutations in 212/1448 (14.6%) CRCs.

### Replication cohort

To replicate findings, we used UKB participant data (under project application number 65833), who were aged between 40 and 69 years at time of recruitment^25^. Germline genotyping of UKB patients was performed using the Applied Biosystems UK BiLEVE Axiom Array by Affymetrix. Briefly, 850,000 SNPs were genotyped and imputed to > 90 million using the Haplotype Reference Consortium^26^, UK10K and 1000 genomes project^27^ reference panels. SNPs were removed if they had INFO scores < 0.8, missingness > 5%, MAF < 5% or HWE exact test^28^ *P* < 1.0 × 10^–6^. We excluded individuals from analysis if they failed one or more of the following thresholds: overall successfully genotyped SNPs < 99% or low heterozygosity (inbreeding coefficient > 0.2), duplication or cryptic relatedness (KING-kinship coefficient > 0.0442 for up to third degree cousins), and evidence of non-white European ancestry by PCA-based analysis. After QC, genotype data was available on 5,078 patients with CRC (assigned by ICD10 code).

### Statistical analyses

We previously identified 11 clinicopathological factors associated with survival in patients from COIN and COIN-B^10^. Due to the number of covariates added to the regression models, dimensionality reduction was performed using PCA to guard against overfitting. A threshold of 70% total variance explained was used to select the number of principal components to include^29^. We carried out GWAS for OS by location of the primary tumour under an additive model. For any SNPs suggestive of an association (*P* < 1.0 × 10^–5^) we performed clumping and tested lead SNPs at independent loci (n = 54) in replication cohorts from the UKB. *P* < 0.05 was used as the significance threshold for replication. Results are reported in accordance with STREGA guidelines.

Power to detect an effect of rs313566 on survival in UKB patients with proximal, distal and rectal tumours was estimated using an additive model, HR = 0.52 (as observed in COIN and COIN-B), *P* = 0.05 and sample sizes of 1433 (473 events), 1450 (420 events) and 1869 (495 events), respectively.

To increase the power to detect associations, we also performed GWAS for survival in UKB patients by location of their colorectal tumour, using age and sex as covariates, followed by genome-wide meta-analysis with the COIN and COIN-B data using a fixed-effects model.

Gene and gene-set analyses: The threshold for significance at gene level was set at *P* < 2.5 × 10^–6^, corresponding to a Bonferroni correction for 20,000 independent tests^30^. Correction for multiple testing for gene-set analysis was made by adjusting *P* values for the false discovery rate (Q < 0.05)^31,32^.

### Bioinformatic analyses

Regional association plots were created using LocusZoom (http://locuszoom.org). PCA, survival analyses and Manhattan/quantile–quantile plots were performed using the psych (https://cran.r-project.org/web/packages/psych/index.html), gwasurvivr^33^ and qqman R (https://www.r-project.org/)^34^ packages, respectively. Meta-analyses were performed using the ‘–meta-analysis’ command in PLINK v1.9^35^.

Gene and gene-set analyses were performed with FUMA^36^ using MAGMA^37^ v1.08 (https://ctg.cncr.nl/software/magma). SNPs were annotated to genes if they were located 35 kilobases before the gene’s transcription zone or up to 10 kilobases after. The SNP-wise and competitive models were used for gene and gene-set analyses, respectively^36^.

eQTL analysis was performed by searching the Genotype-Tissue Expression (GTEx) project database (https://gtexportal.org/home/)^38^ for associations between SNPs and gene expression.

We sought an association between *PI4K2B* expression levels in colorectal tumours and survival in 597 CRC patients from THPA^39^ (https://www.proteinatlas.org/ENSG00000038210-PI4K2B/pathology/colorectal+cancer). RNA-seq data was reported as the median number of fragments per kilobase of exon per million reads (FPKM) generated by The Cancer Genome Atlas. Samples were classified as high expression using a threshold of FPKM > 7.38 as per THPA recommendations^39^. We also performed survival analysis using a linear Cox-proportional hazards model.

## Data availability

The GWAS summary statistics are available through the NHGRI-EBI GWAS Catalog under study accession numbers GCST90250827 (Proximal Colon), GCST90250828 (Distal Colon) and GCST90250829 (Rectum).

## Supplementary Information

Supplementary Information.
