# Supplementary material for: Relationship between inherited genetic variation and survival from colorectal cancer stratified by tumour location
Source: Sci Rep. 2025 Jan 18;15:2423. doi: 10.1038/s41598-024-77870-0 (PMC11742712; doi:10.1038/s41598-024-77870-0)
Supplement: Supplementary file 1 — Supplementary Information. [file 41598_2024_77870_MOESM1_ESM.pdf]

**SUPPLEMENTARY INFORMATION**

# Relationship between inherited genetic variation and survival from colorectal cancer stratified by tumour location

Christopher Wills, Katie Watts, Amy Houseman, Timothy S. Maughan, David Fisher, Nada A. Al-Tassan, Richard S. Houlston, Valentina Escott-Price and Jeremy P. Cheadle.

## SUPPLEMENTARY FIGURES

(A)

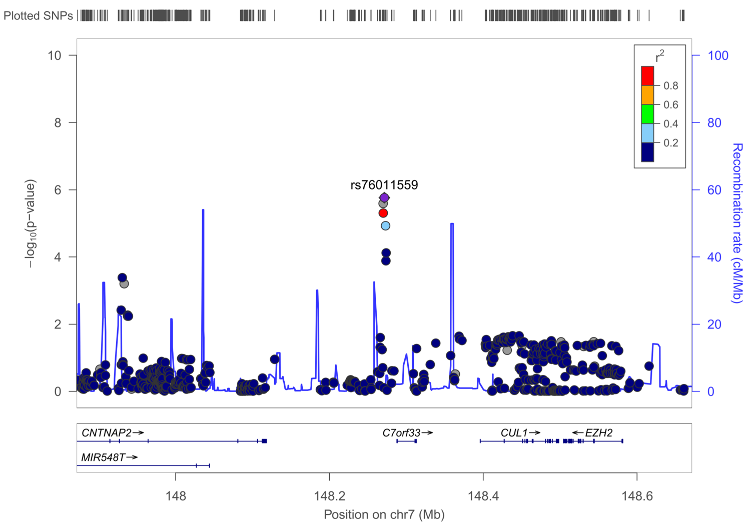


(B)

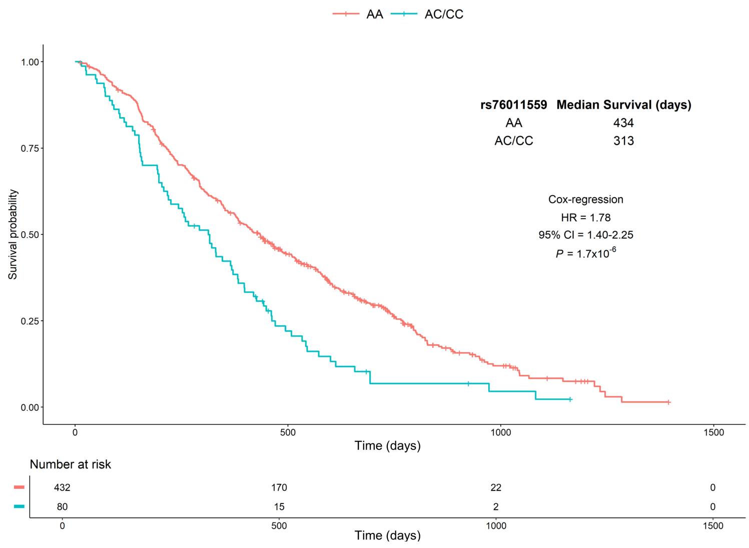


**Supplementary Figure 1. Relationship between rs76011559 genotype and survival in patients from COIN and COIN-B with proximal colon tumours. (A)** Regional locus zoom plot shows results of the analysis for single nucleotide polymorphisms (SNPs) and recombination rates. $-\log_{10}(P)$ (y axis) of the SNPs are shown according to their chromosomal positions (x axis) for an area 400Kb upstream and downstream of rs76011559 (in purple). The colour intensity of each symbol reflects the extent of linkage disequilibrium with the sentinel SNP, deep blue ($r^2$=0) through to dark red ($r^2$=1.0). Genetic recombination rates, estimated using 1000 Genomes Project samples, are shown with a blue line. Physical positions are based on NCBI build 37 of the human genome. Also shown are the relative positions of genes and transcripts mapping to the region of association. Genes have been

redrawn to show their relative positions; therefore, maps are not to physical scale. **(B)** Kaplan-Meier plot of the relationship between rs76011559 genotype and survival. Time in days plotted against survival probability for patients homozygous for the major allele (AA) and heterozygous (AC) or homozygous for the minor allele (CC). The number of patients still at risk at each time point is shown beneath.

(A)

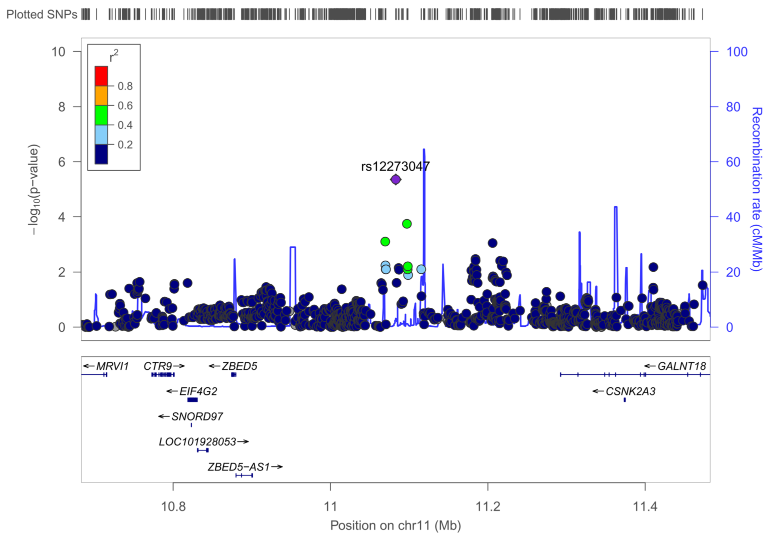


(B)

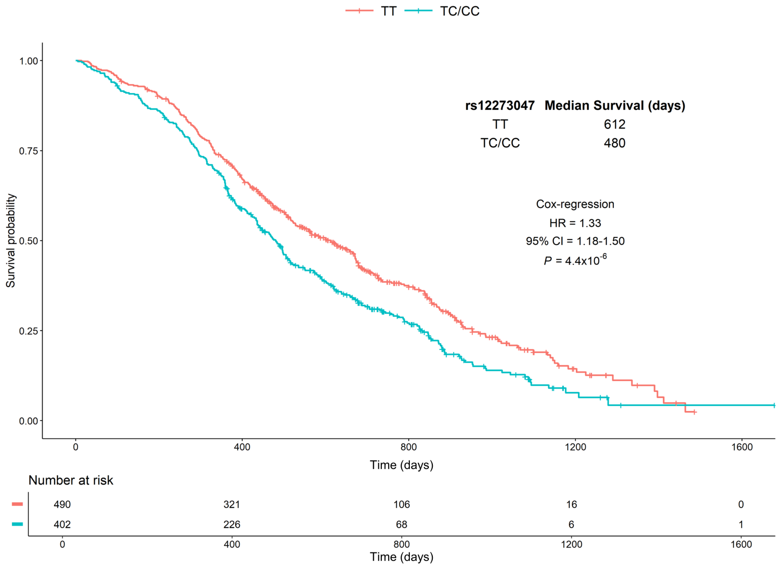


**Supplementary Figure 2. Relationship between rs12273047 genotype and survival in patients from COIN and COIN-B with rectal tumours. (A)** Regional locus zoom plot shows results of the analysis for single nucleotide polymorphisms (SNPs) and recombination rates. $-\log_{10}(P)$ (y axis) of the SNPs are shown according to their chromosomal positions (x axis) for an area 400Kb upstream and downstream of rs12273047 (in purple). The colour intensity of each symbol reflects the extent of linkage disequilibrium with the sentinel SNP, deep blue ($r^2$=0) through to dark red ($r^2$=1.0). Genetic recombination rates, estimated using 1000 Genomes Project samples, are shown with a blue line. Physical positions are based on NCBI build 37 of the human genome. Also shown are the relative positions of genes and transcripts mapping to the region of association. Genes have been redrawn to show their relative positions; therefore, maps are not to physical scale. **(B)** Kaplan-Meier plot

of the relationship between rs12273047 genotype and survival. Time in days plotted against survival probability for patients homozygous for the major allele (TT) and heterozygous (TC) or homozygous for the minor allele (CC). The number of patients still at risk at each time point is shown beneath.

| Location | Hazard ratio | lower 95% CI | upper 95% CI | *P* |
|---|---|---|---|---|
| COIN Distal | 0.52 | 0.40 | 0.70 | $1.8x10^{-7}$ |
| COIN Proximal | 1.10 | 0.89 | 1.36 | 0.37 |
| COIN Rectal | 1.16 | 0.97 | 1.39 | 0.09 |
| UKB Distal | 1.15 | 0.93 | 1.42 | 0.19 |
| UKB Proximal | 1.03 | 0.84 | 1.29 | 0.74 |
| UKB Rectal | 1.11 | 0.91 | 1.34 | 0.29 |

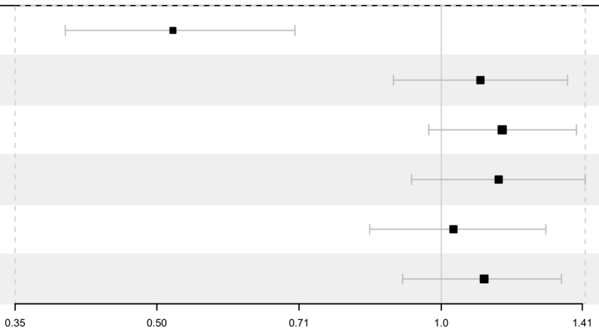


**Supplementary Figure 3: Relationship between rs313566 and survival in patients from COIN and COIN-B, and UKB by tumour location.** Forest plot showing hazard ratio (HR), upper/lower 95% confidence intervals (CI), and *P*-value for survival in patients from COIN and COIN-B (COIN), and the UK Biobank (UKB) carrying one copy of the rs313566 minor (A) allele relative to those homozygous for the major (G) allele.

| Tissue | Samples | NES | p-value |
|---|---|---|---|
| Brain - Cerebellum | 209 | 0.723 | 1.3e-9 |
| Brain - Cerebellar Hemisphere | 175 | 0.588 | 3.8e-5 |
| Spleen | 227 | 0.350 | 1.4e-5 |
| Uterus | 129 | 0.306 | 0.01 |
| Kidney - Cortex | 73 | 0.284 | 0.2 |
| Adrenal Gland | 233 | 0.274 | 2.6e-5 |
| Prostate | 221 | 0.271 | 1.0e-3 |
| Ovary | 167 | 0.270 | 0.007 |
| Nerve - Tibial | 532 | 0.229 | 6.6e-6 |
| Brain - Cortex | 205 | 0.213 | 0.01 |
| Pituitary | 237 | 0.202 | 0.01 |
| Brain - Nucleus accumbens (basal ganglia) | 202 | 0.178 | 0.04 |
| Brain - Spinal cord (cervical c-1) | 126 | 0.155 | 0.2 |
| Brain - Frontal Cortex (BA9) | 175 | 0.144 | 0.1 |
| Skin - Sun Exposed (Lower leg) | 605 | 0.141 | 5.1e-6 |
| Brain - Amygdala | 129 | 0.135 | 0.4 |
| Brain - Substantia nigra | 114 | 0.130 | 0.3 |
| Breast - Mammary Tissue | 396 | 0.130 | 0.03 |
| Stomach | 324 | 0.125 | 0.01 |
| Brain - Anterior cingulate cortex (BA24) | 147 | 0.125 | 0.1 |
| Thyroid | 574 | 0.113 | 0.02 |
| Lung | 515 | 0.110 | 0.03 |
| Brain - Hippocampus | 165 | 0.106 | 0.2 |
| Artery - Aorta | 387 | 0.101 | 0.05 |
| Esophagus - Gastroesophageal Junction | 330 | 0.0942 | 0.1 |
| Muscle - Skeletal | 706 | 0.0817 | 0.09 |
| Liver | 208 | 0.0762 | 0.2 |
| Esophagus - Muscularis | 465 | 0.0749 | 0.2 |
| Pancreas | 305 | 0.0712 | 0.2 |
| Skin - Not Sun Exposed (Suprapubic) | 517 | 0.0695 | 0.05 |
| Brain - Hypothalamus | 170 | 0.0608 | 0.6 |
| Heart - Left Ventricle | 386 | 0.0594 | 0.2 |
| Adipose - Subcutaneous | 581 | 0.0502 | 0.2 |
| Artery - Tibial | 584 | 0.0487 | 0.3 |
| Artery - Coronary | 213 | 0.0427 | 0.6 |
| Small Intestine - Terminal Ileum | 174 | 0.0318 | 0.5 |
| Testis | 322 | 0.0266 | 0.6 |
| Colon - Sigmoid | 318 | 0.0265 | 0.7 |
| Brain - Caudate (basal ganglia) | 194 | 0.0147 | 0.9 |
| Heart - Atrial Appendage | 372 | 0.0123 | 0.8 |
| Whole Blood | 670 | 0.00575 | 0.8 |
| Brain - Putamen (basal ganglia) | 170 | 0.00334 | 1 |
| Adipose - Visceral (Omentum) | 469 | -0.00496 | 0.9 |
| Cells - EBV-transformed lymphocytes | 147 | -0.0226 | 0.9 |
| Colon - Transverse | 368 | -0.0497 | 0.2 |
| Esophagus - Mucosa | 497 | -0.0653 | 0.03 |
| Minor Salivary Gland | 144 | -0.0821 | 0.3 |
| Cells - Cultured fibroblasts | 483 | -0.104 | 2.6e-5 |
| Vagina | 141 | -0.119 | 0.1 |

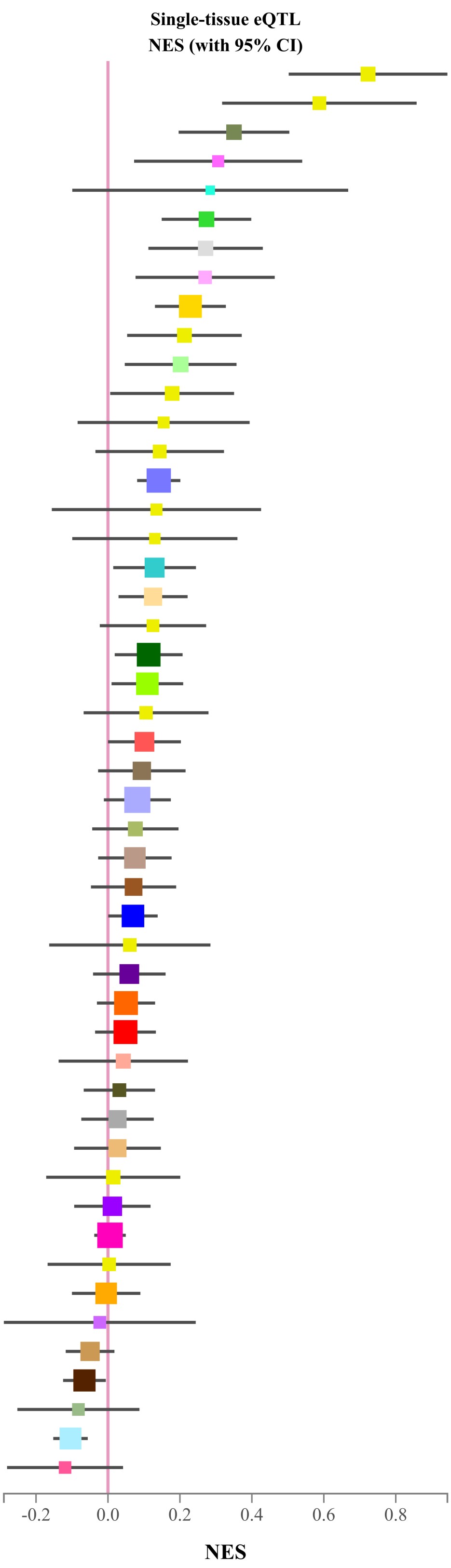


**Supplementary Figure 4: Relationship between rs313566 and expression of Phosphatidylinositol 4-Kinase Type 2 Beta (*PI4K2B*).** rs313566 was an expression quantitative trait loci (eQTL) for *PI4K2B* in several cell types with the A-allele associated with increased *PI4K2B* expression. The GTEx tissue, sample size, eQTL effect size (NES; the

slope of the linear regression of normalised expression data and rs313566 genotype), its associated 95% confidence intervals (CI), and *P*-value are shown.

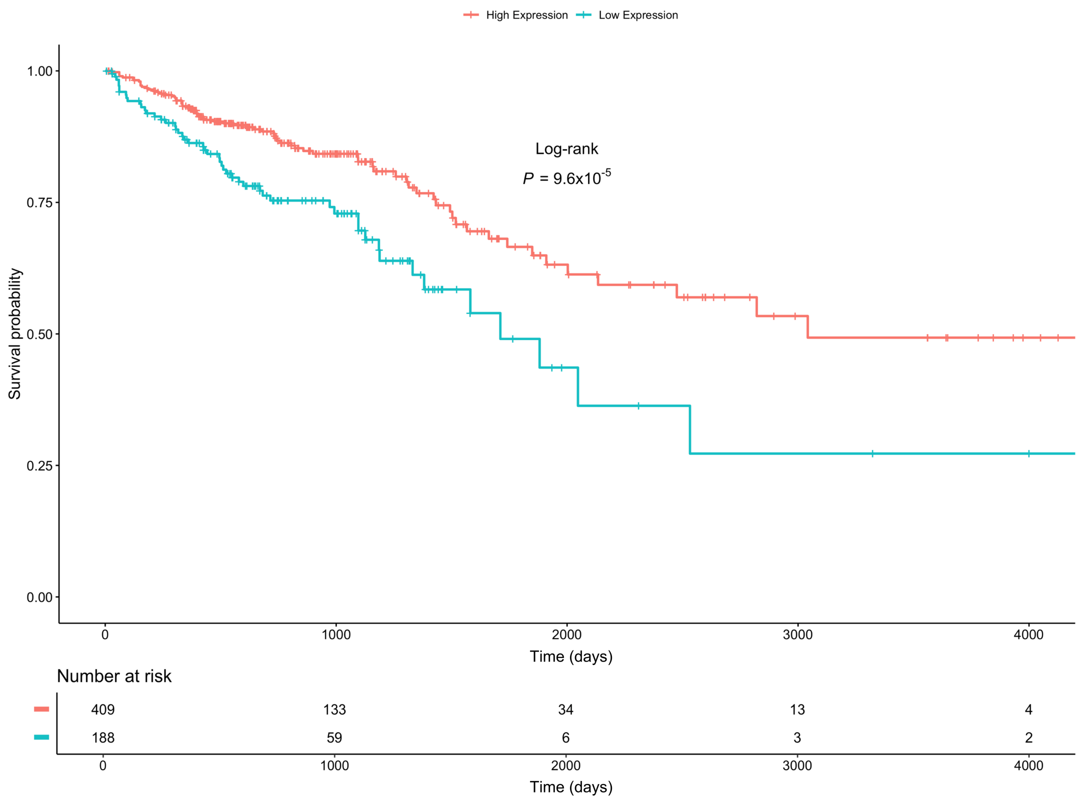


**Supplementary Figure 5. Kaplan-Meier plot for *PI4K2B* expression levels in colorectal tumours from 597 patients from the Human Protein Atlas.** Time in days plotted against survival probability. High expression levels defined as median number of fragments per kilobase of exon per million reads >7.38. A log-rank test was used to calculate *P*-value for differences in survival between the groups.

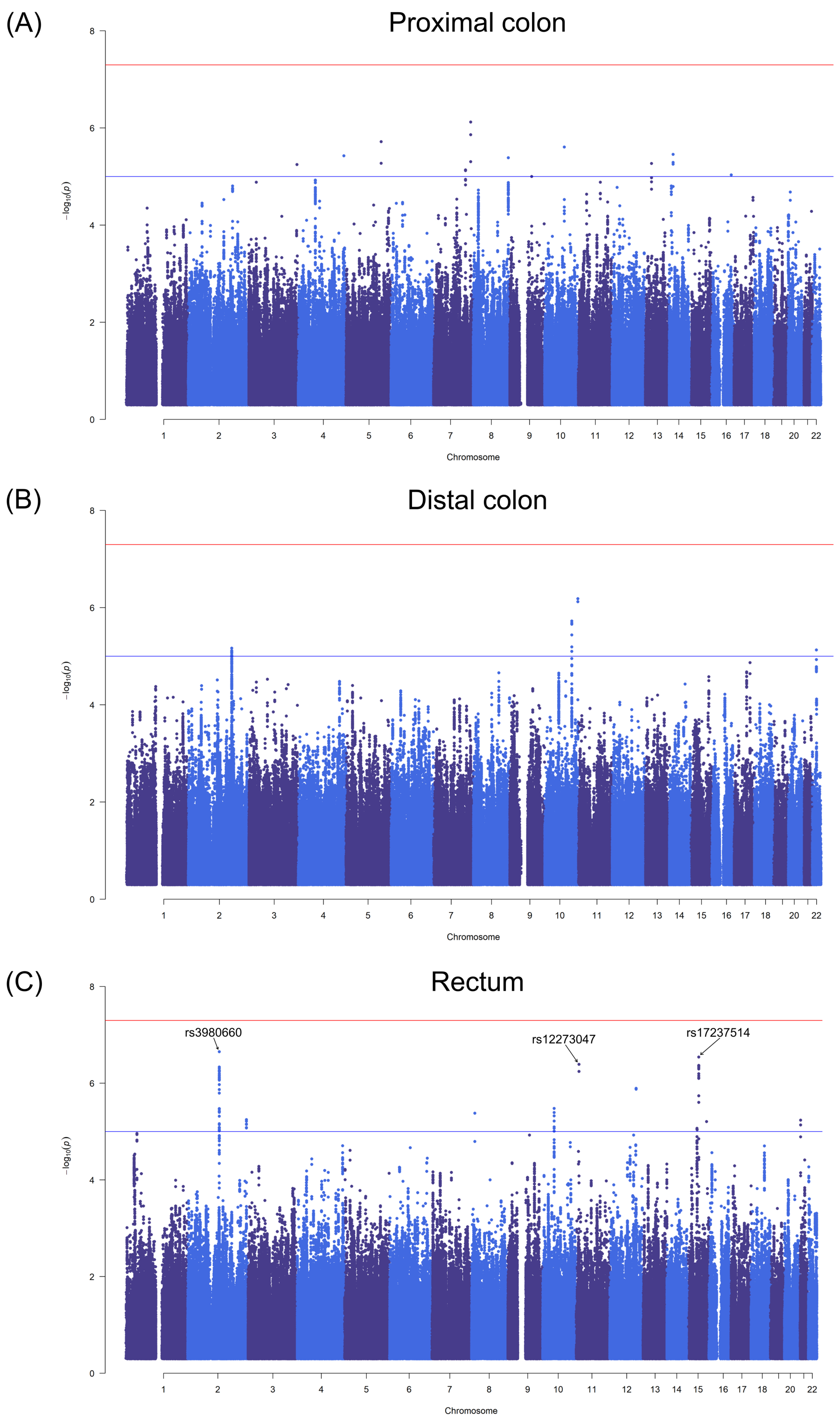


**Supplementary Figure 6. Manhattan plots of single nucleotide polymorphism (SNP) associations with survival from a meta-analysis of CRC patients from COIN, COIN-B and the UK Biobank by primary tumour location.** (A) Proximal colon (n=1,947), (B) distal colon (n=1,943) and (C) rectum (n=2,761, arrows denote rs3980660 at 2q14.3, rs12273047 at 11p15.4 and rs17237514 at 15q22.2 which were close to genome-wide significant thresholds). SNPs are ordered by chromosome position and plotted against the $-\log_{10}(P)$ for

their association with overall survival. The red line represents the threshold for genome-wide significance ($P<5.0\times10^{-8}$) and the blue line is the threshold for suggestive significance ($P<1.0\times10^{-5}$).

## SUPPLEMENTARY TABLES

**Supplementary Table 1. MAGMA gene-set analysis for survival in patients from COIN and COIN-B by tumour location.**

| Primary tumour location | GO Term | Gene-Set Name | *P* | *q* |
|---|---|---|---|---|
| Rectal | GO:0071072 | Negative regulation of phospholipid biosynthetic process | $6.7 \times 10^{-11}$ | $6.6 \times 10^{-7}$ |
| | GO:0006654 | Phosphatidic acid biosynthetic process | $5.6 \times 10^{-7}$ | $2.8 \times 10^{-3}$ |
| | GO:0047184 | 1-acylglycerophosphocholine O-acyltransferase activity | $8.5 \times 10^{-6}$ | $2.8 \times 10^{-2}$ |
| | GO:0007616 | Long term memory | $1.6 \times 10^{-5}$ | $3.9 \times 10^{-2}$ |

Statistically significant sets with $q<0.05$ are presented. Gene-ontology (GO) term, full descriptive name, *P*-value and corrected *P*-value (*q*) are shown.

**Supplementary Table 2. MAGMA gene-set analysis for survival in the meta-analysis of patients from COIN, COIN-B and the UK Biobank by tumour location.**

| Primary tumour location | GO Term | Gene-Set Name | *P* | *q* |
|---|---|---|---|---|
| Rectal | GO:0071072 | Negative regulation of phospholipid biosynthetic process | $9.6 \times 10^{-12}$ | $9.5 \times 10^{-8}$ |
| | GO:0006654 | Phosphatidic acid biosynthetic process | $8.2 \times 10^{-8}$ | $4.1 \times 10^{-4}$ |
| | GO:1905898 | Positive regulation of response to endoplasmic reticulum stress | $1.4 \times 10^{-5}$ | $4.5 \times 10^{-2}$ |

Statistically significant sets with $q<0.05$ are presented. Gene-ontology (GO) term, full descriptive name, *P*-value, and corrected *P*-value (*q*) are shown.
